# A Meta-Regression of Racial Disparities in Wellbeing Outcomes During and After Foster Care

**DOI:** 10.1177/15248380221111481

**Published:** 2022-06-30

**Authors:** Reeve S. Kennedy, Marina H. Potter, Sarah A. Font

**Affiliations:** 1 Department of Sociology and Criminology, 8082 The Pennsylvania State University, University Park, PA, USA; 2 Child Maltreatment Solutions Network, 8082 The Pennsylvania State University, University Park, PA, USA; 3 School of Social Work, East Carolina University, Greenville, NC, USA

**Keywords:** foster care, race, ethnicity, wellbeing, disparities, foster youth

## Abstract

Children in foster care face heightened risk of adverse psychosocial and economic outcomes compared with children in the general population. Yet, the effects of foster care as an intervention are heterogeneous. Heterogeneity outcomes by race and ethnicity are of particular interest, given that Black and Indigenous youth experience foster care at higher rates than other racial/ethnic groups and experience group differences in setting, duration, and exits to permanency. This meta-regression explores racial disparities in education, employment, mental health, and behavioral outcomes during and following foster care. A systematic search of PsycINFO, ERIC, and Academic Search Complete using a series of search terms for studies published between January 2000 and June 2021 found 70 articles and 392 effect sizes that provided outcomes of US-based foster care by race/ethnicity. Findings reveal that Black foster care impacted persons (FCIPs) have 20% lower odds (95% CI: .68–.93) of achieving employment or substantial financial earnings and have 18% lower odds (95% CI: .68–1.00) of mental health concerns compared to White FCIPs. Hispanic FCIPs have 10% lower odds (95% CI: .84–.97) of achieving stable housing compared to non-Hispanic FCIPs. Moderator analyses revealed certain study features (i.e. publication type, timing of the study, location of the study, and placement status of the participants) have a significant impact on the gap between Black and non-Black and Hispanic and non-Hispanic FCIPs. The findings provide important implications for racial disparities in foster care outcomes, as well as highlight important gaps and missing information from published studies.

Foster care provides full-time temporary care for children deemed unable to remain safely with their families of origin, typically as a result of child abuse or neglect. Yet, foster care is a highly disruptive intervention and may not provide adequate compensatory care to mitigate the impacts of child maltreatment. Overall, research suggests heterogeneous––positive, negative, and null—effects of foster care, with the direction and magnitude of effects varying by sample composition, outcome of interest, location of study, methodology, and other factors (Font & Gershoff, 2020a). Consequently, there are growing calls for more research into the heterogeneous effects of foster care (Font & Kennedy, 2022), and particularly the nature and extent of disparate foster care experiences and outcomes by race (Barth et al., 2020).

It is widely established that in the United States, as well as other majority Anglo countries, Black and Indigenous children experience foster care placement at higher rates than white children (Cénat et al., 2021), and typically also at higher rates than Hispanic and Asian children (Yi et al., 2020). This gap has diminished, but not disappeared, for Black children in the United States over the past two decades (U.S. Department of Health and Human Services, 2006, 2020). Thus, the impacts of foster care––whether positive, neutral, or negative–are disproportionately conferred on Black and Indigenous children.

Beyond different rates of exposure, some have argued that foster care is differentially harmful to children of color (UpEnd Movement, 2021). Children of color are more likely than White children to be placed in communities and with caregivers who do not share children’s racial, ethnic, and cultural origins. In the absence of proactive mitigation efforts, foster care may increase their exposure to racism and induce challenges related to identity and self-esteem (Smith et al., 2008). Further, limited research suggests that Black children may encounter less supportive foster care experiences than White children, such as higher rates of sibling separation (Font & Kim, 2021) and running away (Wulczyn, 2020) and greater exposure to group and institutional care (U.S. Children’s Bureau, 2015). Lastly, despite a sufficient number of prospective adoptive families (Karmack et al., 2012), Black and American Indian/Alaska Native youth are subjected to disproportionately longer foster care stays (Huggins-Hoyt et al., 2019) and lower rates of adoption (Akin, 2011; Barth, 1997).

Yet, there is little evidence on whether the health and wellbeing outcomes of children who experience foster care differ by race. In the general population, racial disparities in high school dropout rates have narrowed significantly (Child Trends, 2021), though large and persistent racial disparities among youth in the general population persist in employment (Spievack & Sick, 2019), justice systems involvement (Rovner, 2016), and teen parenthood (Osterman et al., 2021). Foster care may sustain, aggravate, or mitigate those disparities. A recent review article examined racial differences in outcomes following Child Welfare System (CWS) involvement and found little evidence of profound negative effects of CWS involvement, regardless of race, nor substantial racial disparities in outcomes following CWS involvement (Barth et al., 2020). These findings were somewhat limited, as this study used a systematic review format, focused on all types of CWS involvement, and had little data specifically on Black youth. By using a meta-analytic approach, we are able to analyze more studies, including those that utilize race as a control even if they did not explicitly address race in their results. Using a meta-analytic framework, this study assesses racial disparities in health and wellbeing outcomes among children during and after foster care.

## Methods

### Inclusion Criteria

The purpose of this meta-analytic review was to synthesize and analyze results from research studies that reported wellbeing outcomes by race and ethnicity for individuals residing in or previously placed in foster care (inclusive of non-relative family foster care, kinship care, group homes, and residential facilities) via the Child Welfare System. For brevity, we will refer to this subpopulation as foster care-impacted persons, or FCIPs. Included studies were based on the United States foster care population, written in English, and published (in peer review or dissertation format) between January 2000 and June 2021. We included studies of participants currently in foster care as well as studies of children or adults with prior foster care experience (e.g., youth aging out of care). In addition, in order to assess racial disparities in outcomes, included studies needed to provide disaggregated (separately reported) data on two or more racial or ethnic groups. We concentrated on outcomes of “foster care as usual,” and thus excluded studies that focused exclusively on intervention programs or treatment foster care, unless we were able to discern outcomes for a “care as usual group.” Relatedly, we excluded samples that were recruited or limited based on specific characteristics (e.g., juvenile justice involvement, developmental disabilities, high-risk, emotionally disturbed, or homeless populations). We did not restrict studies based on research design or methodological quality (Lipsey & Wilson, 2001). In addition to peer-reviewed literature, we included reports and dissertations in our systematic search to reduce publication bias. The meta-analysis protocol was submitted to PROSPERO.

### Identification of Studies

To locate all studies that addressed outcomes of foster care by race/ethnicity, the lead author conducted a systematic search of PsycINFO, ERIC, and Academic Search Complete using a series of search term combinations. These search terms addressed the type of placement (e.g., “foster care,” “kinship care,” “out of home care”) and the type of outcome (e.g. “behavior* problem” or “externalizing” or “internalizing,” “mental health” or “depress*”). For more information about the specific terms and combinations, see Appendix B. We chose not to include race/ethnicity terms in the search criteria because that would likely omit studies that considered race/ethnicity as a covariate or descriptive indicator rather than a main focus of the analysis. We sought to include studies that report outcomes by race/ethnicity or include race/ethnicity as a control variable regardless of whether they highlighted race/ethnicity in keywords, titles, or abstracts.

**Figure 1.**
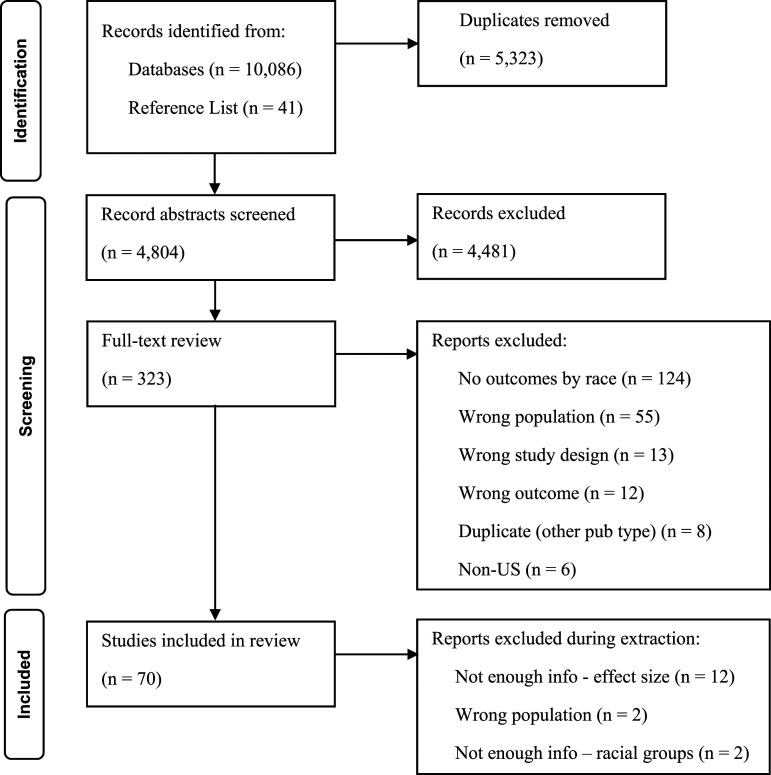
Flow chart of systematic search.

Figure 1 presents a flow chart of the search process and the reasons for exclusion during full-text review. The search yielded 10,086 studies, with 5,323 removed as duplicates. Forty-one additional studies were added from the reference list of a related review article (Barth et al., 2020). Abstracts were reviewed for 4804 studies by the lead author using Rayyan QCI. After abstract review, 323 studies were included for full-text review. Full-text review was conducted independently by the first and second authors using Rayyan QCI. Conflicts were reviewed and discussed. The final analytic sample included 70 studies with 392 effect sizes. For a summary table of included studies, see Appendix A–Table 1.

### Data Extraction and Coding Process

A comprehensive coding scheme was developed a priori using previous research as a guide (see Appendix B for more details about study selection and coding, including the coding scheme). Data extraction was completed by the first and second authors. Extracted data were compared and disagreements were discussed until agreement was reached.

#### Effect sizes

For included studies, effect size information was extracted for each relevant outcome, including the groups being compared, the outcome measure, the type of effect size (e.g., odds ratio, mean difference), and whether the effect size was adjusted (coefficient from a multivariate model) or unadjusted (bivariate comparison of means or proportions, or unadjusted odds ratios). The primary effect size measures were odds ratios. When studies reported outcomes using other measures (e.g., correlation, means, regression coefficients), the effect size was converted to an odds ratio using the Campbell Effect Size Calculator (Wilson, n.d.). Considering race/ethnicity is frequently included in studies as a control variable, we found it prudent to include regression coefficients. These coefficients were converted to Cohen’s D, then to odds ratios. This is a widely accepted approach in the literature (Aloe & Becker, 2012; Hunter & Schmidt, 2004; Tsaousis, 2016). When extracting effect sizes from regression analyses, we used the most complete model available. Effect sizes were grouped into domains by outcome measure: educational achievement, education/employment, education/earnings, homelessness, high risk and externalizing behaviors, mental health concerns, and justice system involvement. In addition, we created two pooled domains: positive outcomes and negative outcomes. For the positive effect sizes (educational achievement, education/employment, and education/earnings) a higher score represents a more positive outcome, and for the negative effect sizes (homelessness, high risk and externalizing behaviors, mental health concerns, and justice system involvement) a lower score represents a more positive outcome. Additional information on the measures included within each domain is found in Table 1.

**Table 1.** Domain Details.

Domain	*k*	*n*
Collapsed Indicators		
Educational achievement	28	120
Degree/educational attainment	21	93
Test scores or GPA	7	26
School attendance	1	1
Education/employment	2	7
Employment or degree/educational achievement	2	7
Employment/earnings	16	53
Employment	15	36
Material hardship	1	1
Public assistance receipt	3	9
Income level	3	6
Health insurance	1	1
Homelessness	11	32
Home ownership	2	2
Homelessness	10	27
Housing insecurity	1	3
High risk and externalizing behaviors	25	77
Externalizing behaviors/delinquency	17	43
Risky sexual behavior	3	8
School behavioral problems	4	8
Substance abuse	10	18
Mental health concerns	27	69
Internalizing symptoms	21	43
Mental health disorder diagnosis	6	14
Mental health services	4	12
Justice system involvement	14	34
Adult corrections	2	8
Arrest	4	6
Incarceration	6	15
Juvenile corrections	4	5

k = studies; n = effect sizes.

#### Study and sample attributes

In addition to effect sizes, extracted data elements included study characteristics (publication type, study design, sampling method, data source, data type, region of data collection, date of baseline data collection, and date of outcome data collection) and sample attributes (independent living services, modal placement setting, age at entry, average length of stay, average number of placements, type of exit, and mean age at outcome). Due to substantial missingness on many of these attributes, not all were able to be considered as potential moderators of effect size. Ten covariates were considered in the meta-regression analysis: *baseline year* (0 = < 2002, 1 = 2002+) refers to the first year of baseline data collection and *outcome year* (0 = <2008, 1 = 2008+) refers to the first year of the outcome data collection. These cutoff points represent the approximate median years at both the study level and effect size level. In addition, there have been shifts in policies for and the demographics of foster care since the late 1990’s/early 2000’s (Font & Kennedy, 2022), so using a cut-off date from the early 2000’s helps capture those shifts. *Survey random* refers to studies that used survey methods and random sampling, as opposed to administrative data (survey random = 1, administrative = 0). *Survey non-random* refers to studies that used survey methods but did not have a random sample, as opposed to administrative data (survey non-random = 1, administrative = 0). *Effect size type* refers to the type of effect size data that was extracted from each study (regression coefficient = 1, other type = 0). *National* refers to whether the sample was nationally representative (national = 1). *Out of care adult* refers to whether the participants were out of care and over 18 at the time of outcome data collection, as opposed to participants who were still in care (out of care adult = 1, in care = 0). *Out of care––minor* indicates whether the participants were out of care and under age 18, as opposed to participants who were still in care (out of care minor = 1, in care = 0). *Self-report* refers to whether the source of the data was self-report (self-report = 1). Lastly, we included an indicator for *peer-review* (peer-reviewed publication = 1, dissertation = 0).

### Statistical Analysis

We conducted random effects meta-regressions using robust variance estimation (RVE) to calculate summary effects for each racial group comparison within six^1^ outcome domains, then we employed a moderator analysis to assess the impact of select study and sample level predictors. Random effects meta-regressions were used because we expect there to be heterogeneity in effect sizes due to differences across studies and samples, this is in contrast with a fixed effects meta-regression that expects more homogenous effect sizes (Borenstein et al., 2009). RVE adjusts models to account for correlation both within and between studies (Hedges et al., 2010), which allowed us to include as many relevant studies as possible, despite repeated datasets among studies––six out of 39 datasets were used by more than one study^2^––and multiple effect sizes per study. Extracted effect sizes pertain to specific racial group comparisons (e.g., Black vs. White). Due to the small number of studies examining other racial and ethnic groups, we focus our analysis on the following comparisons: Black-White; Black–non-Black, Hispanic–White; and Hispanic–non-Hispanic. Separate meta-regressions were conducted for each comparison group within each outcome domain. We used inverse variance weights with Hedges variance estimation (Hedges et al., 2010).

First, we produced intercept-only models, which provide the average weighted effect size for each comparison group within each domain (Tanner-Smith & Tipton, 2014). Second, multivariate RVE meta-regressions were used to consider study and sample attributes as potential moderators. Because meta-regression requires a minimum of 10 effect sizes per covariate (Borenstein et al., 2009), we could only examine one covariate at a time. All meta-regressions were conducted in Stata using *Robumeta* (Hedberg, 2014). In *Robumeta*, due to correlated effect sizes, it is necessary to identify a within-study effect size correlation value*,* we used RHO (.08). *Robumeta* works best when there are a minimum of 40 studies included in the meta-analysis with five effect sizes per study (Tanner-Smith & Tipton, 2014). However, *Robumeta* includes a default small sample adjustment that adjusts both the estimator and the degrees of freedom (DF) (Tipton, 2015), thus improving the estimate and *p*-value for samples that include fewer than 40 studies, which is very common in meta-analysis. Despite the small sample adjustment, some of our models had DF < 4, which is likely due to imbalances in the data, so these were noted. When DF < 4, the incidence of Type 1 error is likely underestimated (Tanner-Smith et al., 2016; Tipton, 2015), so the *p*-values need to be interpreted with caution. In these instances we used a conservative *p <* .001 to indicate significance (Tanner-Smith et al., 2016; Tanner-Smith & Tipton, 2014). Relatedly, if the number of effect sizes per domain and comparison group was less than 10, they were not included in the covariate analysis. Similarly, if the events per variable (EPV) for a covariate was less than five within a given domain/comparison group then we did not include them in our covariate analysis (Vittinghoff & McCulloch, 2007).

### Sensitivity Tests and Publication Bias

We conducted a series of sensitivity tests to assess the robustness of our analysis, all of which had no significant impact on our primary findings. The first set of sensitivity tests involved testing different within-study correlation values in our RVE meta-regression. Our primary analyses used RHO (.08): for the sensitivity tests we ran all analyses with RHO (.07) and RHO (.09). This approach is consistent with previous literature (Collins et al., 2018; Pham et al., 2021; Tanner-Smith & Tipton, 2014). The second set of sensitivity tests assessed the impact of 20 effect sizes that were highly correlated (measured the same or extremely similar constructs with the same sample) with other within-study effect sizes. These tests also had no impact on our primary findings. Publication bias was assessed using funnel plots and egger regressions tests for small study effects. Funnel plots identify asymmetry in effect sizes, which may reflect heterogeneity rather than or in addition to publication bias (Sterne et al., 2011), while the egger regression test is a linear test used to determine if smaller studies show different effects than larger studies (Egger et al., 1997; Sterne et al., 2011). For more details and results from the sensitivity tests and tests for publication bias, see Appendix C.

## Results

### Attributes of Included Studies

The systematic search yielded 395 effect sizes across 70 studies using 39 unique datasets. Study and sample characteristics are displayed in Table 2. The included studies began baseline data collection between 1990 and 2014, with an average start year of 2003, and outcome data collection began between 1996 and 2018, with an average around 2008 (see Table 2 for descriptive features). The majority of the studies were longitudinal (71%), published in peer-reviewed academic journals (76%), used self-report (57%), and survey methods with non-random sampling (47%). Samples were collected from across the US, with many coming from the Midwest (33%) or nationally representative (27%). Very few samples came exclusively from the Northeast (3%) or the South (6%).

**Table 2.** Study Level Descriptive Features (*k* = 70).

Study features	*k*	%/m (range)	Sample features	*k*	%/m (sd)	Range
Unique datasets	39		Race/ethnicity			
First baseline year	61	2003 (1990–2014)	White	69	732 (1,192)	3–7,399
First outcome data year	63	2008 (1996–2018)	Black	62	594 (927)	15–4,756
Publication type			Hispanic	48	414 (1,032)	3–6,832
Dissertation	17	25%	Asian	8	116 (176)	1–402
Peer-reviewed	53	76%	Multi-racial	17	126 (152)	2–503
Region of U.S.			NH/PI^a^	5	6.8 (3.42)	1–10
Northeast	2	3%	AI/NA^b^	14	95 (101)	2–321
South	4	6%	Unknown	13	87 (142)	1–516
Midwest	23	33%	Sex			
West	9	13%	Male	68	794 (1,340)	0–6342
National	19	27%	Female	68	940 (1,522)	35–7100
Multi-region	12	17%	Age	67	18.2 (7.96)	5–42
Unknown	1	1%	Independent living services provided	37	53%	
Data and sampling type			Status at outcome			
Administrative	18	26%	In care	25	36%	
Survey-random	33	47%	Out of care-Minor	7	10%	
Survey––Non-random	19	27%	Out of care-Adult	35	50%	
Data source			Mixed	3	4%	
Self-report	40	57%	Majority placement			
Other	30	43%	Congregate care	5	7%	
Type of effect size			Kinship care	10	14%	
Regression coefficient	38	54%	Non-relative FC	15	21%	
Other	32	46%	Unspecified FC	40	57%	
Study design			Age of entry	19	9.76 (2.51)	1–13.5
Cross-sectional	20	29%	Avg. Length of stay (years)	20	5.23 (3.89)	1.28–13.93
Longitudinal	50	71%	Avg. # of placements	38	4.67 (2.15)	1–10.83
			Age out sample^c^	34	46%	

k = the number of studies.

FC = Foster Care.

^a^Native Hawaiian/Pacific Islander.

^b^American Indian/Alaska Native.

^c^This refers to studies whose sample was almost exclusively “age out” and it overlaps considerably with the Out of Care––Adult category (89% are “age out” samples).

Nearly all included studies had White (*k* = 69) and Black (*k* = 62) participants, and most included Hispanic (*k* = 48) participants. Despite an in interest in other racial groups, in particular American Indian and Indigenous populations, other groups were under-represented in the data, likely due to factors such as small total population size and geographic concentration in select states or regions. Only 14 studies reported outcomes for American Indian or Alaska Native youth, and these outcomes were spread thinly across domains.

Fifty percent of studies were based on youth who were over the age of 18 and no longer in foster care at the time of outcome data collection; most of these were studies of youth who aged out of foster care (89%). Few studies focused on the outcomes of children who exited foster care to reunification (*n* = 4) or adoption (*n* = 1). Thirty-six percent of studies were based on youth in foster care at the time of outcome measurement. Despite the wide variability in and importance of characteristics of the foster care experience, such as age at entry, placement settings, and stability or mode of discharge, many studies did not include this information.

### Summary Effects

Results of the intercept-only RVE meta-regressions on four racial group comparisons across six of the domains are displayed in Table 3. We found no evidence of significant differences by race or ethnicity for educational achievement, high-risk and externalizing behavior, or justice system involvement. However, Black FCIPs had 20% lower odds (OR: .80, 95% CI: .68–.93, *p* < .05) of part-time or full-time employment or substantial earnings compared to White FCIPs. We also found that Black FCIPs had 18% lower odds (OR: .82, 95% CI: .68–1.00, *p* < .05) of experiencing mental health concerns, such as internalizing symptoms, mental health diagnosis, or receipt of mental health services when compared to White FCIPs, and 16% lower odds (OR: .84, 95% CI: .73–.98, *p* < .05) of experiencing mental health concerns compared to non-Black (this combines all racial comparisons including White) FCIPs. Lastly, Hispanic FCIPs had 10% lower odds (OR: .90, 95% CI: .84, .97, *p* < .05) of homelessness than non-Hispanic FCIPs. No other domains revealed significant differences for Hispanic and non-Hispanic FCIPs.

**Table 3.** Overall Summary Effect Sizes by Domain and Comparison Group.

Achievement outcomes	*n*	*k*	OR	95% CI		df	*t* ^2^
				UL	LL		
Education							
Black versus White	39	18	0.96	0.82	1.13	14.9	0.07
Hispanic versus White	24	13	0.98	0.80	1.20	9.53	0.07
Black versus Non-Black^a^	45	20	0.98	0.85	1.13	16.15	0.06
Hispanic versus Non-Hispanic^b^	28	16	0.98	0.84	1.15	11.8	0.05
Employment/earnings							
Black versus White	16	9	**0.80**	0.68	0.93	5.96	0.02
Hispanic versus White^c^	7	6	0.98	0.81	1.21	3.28	0.01
Hispanic versus Non-Hispanic	9	7	0.98	0.82	1.20	3.73	0.01
Homelessness							
Black versus White	9	8	1.08	0.91	1.28	6.54	0.03
Hispanic versus White^c^	6	6	0.90	0.83	0.97	3.04	0
Hispanic versus Non-Hispanic	8	8	**0.90**	0.84	0.97	4.09	0
Behavioral outcomes							
High risk and externalizing behavior							
Black versus White	17	11	1.02	0.79	1.32	8.6	0.09
Hispanic versus White	14	9	0.80	0.56	1.14	6.76	0.08
Black versus non-black	39	19	1.05	0.87	1.27	15.73	0.1
Hispanic versus Non-Hispanic	17	11	0.80	0.61	1.07	7.51	0.05
Mental health concerns							
Black versus White	26	17	**0.82**	0.68	1.00	14.67	0.09
Hispanic versus White	10	9	0.90	0.60	1.36	7.75	0.18
Black versus non-black	32	22	**0.84**	0.73	0.98	18.57	0.07
Hispanic versus Non-Hispanic	11	10	0.90	0.61	1.34	8.22	0.18
Justice system involvement							
Black versus White	9	8	1.16	0.83	1.60	6.72	0.07
Hispanic versus White	7	7	1.06	0.88	1.30	4.01	0.02
Black versus non-black	13	11	1.23	0.95	1.32	8.26	0.07

*n =* # of effect sizes; k = # of studies; OR = odds ratio; CI = confidence interval; LL = lower limit; UL = upper limit; df = degrees of freedom; t^2^ = between-study variance.

Bolded: Statistically significant.

^a^Black versus Non-Black combines all racial comparisons that looked at Black versus another racial group, this includes Black versus White.

^b^Hispanic versus Non-Hispanic combines all racial comparisons that looked at Hispanic versus another racial group, this includes Hispanic versus White.

^c^Degrees of Freedom <4, - significance level for df<4 is *p* < .001.

### Study Features Meta-Regression

To gain a better understanding of how study level factors impact racial comparisons across the domains, we performed a moderator analysis. We conducted individual RVE meta-regressions for Black versus non-Black and Hispanic versus non-Hispanic comparisons across five domains (educational achievement, high risk and externalizing behaviors, mental health concerns, and pooled negative and positive outcomes). These domains were selected because they had a minimum of 10 effect sizes within each domain/racial comparison combination. To ensure that data imbalances did not impact the interpretation of our findings, we excluded all models where the events per variable (EPV) was less than five. The coefficients from the meta-regressions are presented in Appendix D. The odds ratios discussed in this section represent the direction and magnitude of the gap between Black and non-Black FCIPs and between Hispanic and non-Hispanic FCIPs compared to studies without the identified feature.

For ease of interpretation, in addition to the meta-regression coefficients and 95% confidence intervals, we present predicted effect sizes (ORs) for coefficients with statistical significance at *p* < .05. Specifically, we calculated the average odds ratio reported in studies for each category of the study-level predictor variable. For example, we report the average odds ratio for the association between Black (vs. non-Black) race and mental health concerns in studies that began baseline collection on or before 2002 and for studies that began baseline data collection after 2002.

**Figure 2.**
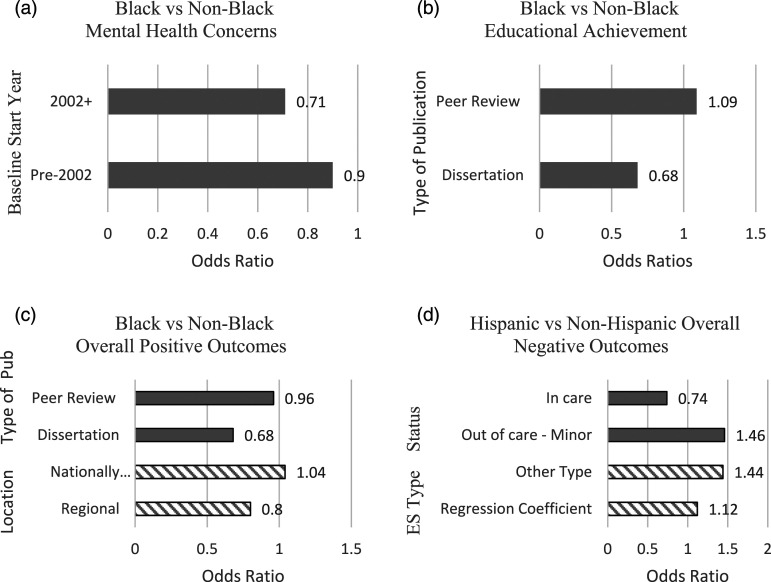
Significant Predictors by Domain and Comparison: Predicted estimates in Odds Ratios. (a) Predicted odds ratios for Black versus Non-Black on Mental Health Concerns by *baseline start year*: pre 2002 and 2002+. (b) Predicted odds ratios for Black versus Non-Black on Educational Achievement by *type of publication*: peer reviewed journal article and dissertation. (c) Predicted odds ratios for Black versus Non-Black on Overall Positive Outcomes by *type of publication*: peer reviewed journal article and Dissertation and by *sample location*: regional and nationally representative. (d) Predicted odds ratios for Hispanic versus Non-Hispanic on Overall Negative Outcomes by *extracted effect size (ES) type*: regression coefficient and other type and by *placement type at outcome*.

Four covariates significantly moderated the gap between Black and non-Black FCIPs: studies published after 2002 compared to those published before 2002 in the mental health domain (OR: .79, 95% CI: .64–.97), peer-reviewed studies compared to dissertations in the educational achievement (OR: 1.60, 95% CI: 1.15–2.23) and positive outcomes (OR: 1.42, 95% CI: 1.02, 1.97) domains, and nationally representative compared to regional studies also in the positive outcomes domain (OR: 1.30, 95% CI: 1.02, 1.63). For the predicted effect sizes, studies that began baseline data collection in or after 2002 found that Black FCIPs had 29% lower odds (mean OR: .71) of mental health concerns compared to non-Black FCIPs, whereas studies that began baseline data collection prior to 2002 found that Black FCIPs had only 10% lower odds (mean OR: .90) of mental health concerns compared to non-Black FCIPs (See Figure 2a). On average, peer-reviewed journals reported slightly more favorable educational outcomes for Black FCIPs than non-Black FCIPs (mean OR = 1.09) whereas dissertations reported substantially less favorable educational outcomes for Black FCIPs (mean OR = .68). Similarly, dissertations found that Black FCIPs had lower odds of positive outcomes (mean OR: .68) than non-Black FCIPs whereas peer reviewed articles reported roughly equal odds of overall positive outcomes (mean OR: .96) (Figures 2b and 2c). In addition, studies that used a nationally representative sample reported similar odds of positive outcomes for Black FCIPs and non-Black FCIPs (mean OR: 1.04) whereas studies with a regional sample found lower odds of positive outcomes among Black FCIPs than non-Black FCIPs (mean OR: .80).

Two covariates significantly moderated the gap between Hispanic and non-Hispanic FCIPs for the negative outcomes domain: studies with regression adjusted effects sizes compared to unadjusted effect sizes (OR: .78, 95% CI: .61–.99) and studies whose participants were out of care and under age 18 compared to studies whose participants were in care (OR: 1.97, 95% CI: 1.43, 2.75). Studies reporting regression-adjusted effect sizes found smaller differences in negative outcomes for Hispanic and non-Hispanic FCIPs (mean OR: 1.12) than studies reporting unadjusted effect sizes (mean OR: 1.44). That is, studies that included covariates found more similar levels of negative outcomes than studies just reporting bivariate group differences. Finally, studies whose participants were out of care and under age 18 at the time of outcome data collection reported higher odds of negative outcomes for Hispanic versus non-Hispanic FCIPs than studies where participants were still in care at the time of outcome data collection. Specifically, estimates suggest that studies of participants who were out of care but still under 18 found that Hispanic FCIPs had 46% *higher* odds (mean OR: 1.46) of negative outcomes compared to non-Hispanic FCIPs, while studies were still in out-of-home care found that Hispanic FCIPs had 36% *lower* odds (mean OR: .74) of negative outcomes compared to non-Hispanic FCIPs.

## Discussion

This meta-regression study sought to characterize the nature and magnitude of racial disparities in various wellbeing domains among foster care involved persons (FCIPs). As concerns about the persistent overrepresentation of Black and Indigenous youth in the US foster care system have risen to the forefront (Dettlaff et al., 2020) following increased public attention to racial inequalities in various social institutions, there is a need for more assessment of the nature and extent of racial disparities in the *outcomes* of children who have experienced child welfare systems involvement (Barth et al., 2020). Foster care, though affecting a small proportion of all CWS-involved persons (Putnam-Hornstein et al., 2021), was the focus of this analysis because it is among the most intensive and controversial interventions that CWS provides. Our analysis reveals substantial limitations of the extent research to adequately assess racial disparities in foster care outcomes. First, the proportion of studies focused on the experiences of individuals who have or are about to age out of foster care is misaligned with the comparatively small proportion of children and youth with foster care experience who age out. Groundbreaking work detailing the hardships of youth aging out (i.e., the Midwest Study) led to large-scale and sustained public investment in providing youth who age out with extended time to prepare for adulthood, free college tuition, health insurance, and other crucial resources. Despite these contributions, research on youth aging out provides relatively little insight into the more than 90% of FCIPS who do not age out of care (U.S. Department of Health and Human Services, 2020). The body of research on wellbeing outcomes following reunification, adoption, and guardianship is quite small (Font & Gershoff, 2020b; Font & Kennedy, 2022), and the number of studies evaluating racial disparities in post-discharge outcomes is smaller still. A majority of children who exit foster care either return to their biological parents or reside permanently with a biological relative (U.S. Department of Health and Human Services, 2020). Given large and persistent racial disparities in neighborhood disadvantage (Reardon et al., 2015), income and wealth, and family structure (McLanahan & Percheski, 2008), racial disparities in outcomes may be larger after discharge than within foster care as children are placed in environments with vastly different resources (Font et al., 2021). Our analysis does find some evidence that gaps in negative outcomes are larger for Black FCIPs than White FCIPs in studies of minors who have exited foster care than in studies of individuals in foster care at the time of outcome measurement, but it is based on a small number of studies.

Second, the nature of foster care varies by child and family circumstances, place, and time and thus, unsurprisingly, estimated effects of foster care on various aspects of wellbeing vary too (Font & Kennedy, 2022). Many of the largest and most troubled foster care systems––which disproportionately placed Black children and youth––drastically reduced both entry rates and length of stay in 2000’s (U.S. Department of Health and Human Services, 2013) and the US system as a whole has seen increases in adoptions and guardianships, decreases in congregate (group/institutional) care, and younger age at entry to care (U.S. Department of Health and Human Services, 2006, 2020). Because racial/ethnic groups are not evenly distributed across the US, the agencies (which are typically county-based) vary in these foster care dynamics, children of different racial/ethnic group may experience widely different foster care experiences depending on where they live and when they come into care. Yet, many studies did not report information on aspects of the foster care experience that are widely thought to be pertinent for child development, such as age at entry, placement stability, type of placement, or length of time spent in foster care. Our analysis suggests that time and place of data collection appear to moderate racial disparities for some outcomes, but it is plausible––likely even––that time and place are capturing unmeasured factors related to the quality and nature of foster care. Additional research is needed to understand the role of such factors on the direction and magnitude of racial disparities in wellbeing during and after foster care.

Third, we note the lack of studies that report outcomes for Indigenous and Asian and Pacific Islander FCIPs. Indigenous children experience comparatively high rates of foster care entry (Yi et al., 2020) and they can be subject, under the Indian Child Welfare Act (1978), to different policies and practices that may result in different types and durations of foster care, and consequently different wellbeing outcomes.

Notwithstanding the limitations of the existing literature, we highlight three findings of interest. There were no consistent gaps in educational outcomes, but Black FCIPs had lower average employment/earnings than non-Black FCIPs. This is consistent with general population research identifying closing gaps in educational outcomes but persistent gaps in employment, the latter of which may be sustained by broader societal problems of unchecked employment discrimination (Quillian et al., 2017) and the lack of employment opportunities for individuals with criminal records (Pager, 2003). That is, in some ways, we observe that racial disparities in outcomes for FCIPs track or mirror patterns in the general population. Yet, we did not find higher rates of either justice system involvement or homelessness among Black and Hispanic FCIPs relative to White FCIPs (and instead found lower rates of homeless for Hispanic FCIPs), despite the large gaps in these experiences within the general population (Fusaro et al., 2018; Wildeman, 2009). This likely reflects that foster care experience, homelessness, and justice systems involvement are concentrated among the socioeconomically disadvantaged, such that the social contexts of Black, White, and Hispanic FCIPs are more similar to one another than are those of Black, White, and Hispanic members of the general population. Table 4 summarizes the critical findings and Table 5 summarizes the implications for practice, policy and research.

**Table 4.** Critical Findings.

1. This analysis included 350 effect sizes from 70 studies with 39 unique datasets. This means that there is a lot of overlap in samples when examining foster care outcomes.
2. Almost one third of the studies focused exclusively on youth who “aged out” of foster care. Though an important subpopulation, aging out is a rare experience among foster youth overall and heavily concentrated among youth who entered foster care as teenagers.
3. Many studies did not report important details of participants’ foster care experiences, such as the length of time in foster care, age at entry, type of exit, or number and type of foster care settings.
4. Despite having the highest rates of foster care placement of all racial/ethnic groups, only 14 studies included American Indian/Alaska native former foster youth, which when distributed across domains were too few to include in our analysis.
5. We found no significant differences in outcomes by race for educational achievement, involvement in high risk or externalizing behaviors, or justice system involvement.
6. Black former foster youth have lower odds of achieving full or part time employment and financial stability compared to White and non-black former foster youth. In addition, black former foster youth have lower odds of mental health concerns than White former foster youth.
7. Hispanic former foster youth are at slightly lower risk of homelessness than non-Hispanic former foster youth.
8. Studies that collected baseline data after 2002 reported lower odds of mental health concerns for black versus non-black former foster youth than studies that started baseline data collection before 2002.
9. Dissertations studies reported significantly lower odds of education achievement or positive outcomes for black versus Non-Black former foster youth compared to peer-reviewed journal articles.
10. Studies with participants who were under age 18 and out of care at the time of outcome data collection reported significantly higher odds of negative outcomes for Hispanic versus non-Hispanic foster youth than studies whose participants were still in care at the time out outcome data collection.
11. Studies that controlled for covariance found lower odds of negative outcomes for Hispanic versus Non-Hispanic former foster youth compared to studies that did not control for covariance.

**Table 5.** Implications for Practice, Policy, and Research.

1. A disproportionate number of studies focus on outcomes of youth who “age out” of foster care––this provides little insight into outcomes following the more than 90% of FCIPs who leave care via other pathways, such as reunification and guardianship. More research is needed regarding foster care outcomes following other exits to permanency.
2. Many studies do not include vital information regarding the varying foster care experiences. Given the widespread variation in foster care experiences and racial demographics of foster care systems, it is possible that some of the differences captured in this study are related to differences in the quality and nature of foster care. Future research studies need to make sure to include details about the foster care experiences of the sample. In addition, more research is needed to understand the role of varying foster care factors on the magnitude and direction of racial disparities in wellbeing during and after foster care.
3. The racial disparities in educational and employment/earnings outcomes largely mirror those of the general population, suggesting that foster care is neither exacerbating nor improving these outcomes.
4. The lack of significant racial disparities in justice system involvement and homelessness is in contrast with the substantial disparities found in the general population and likely reflects that FCIPs tend to come from similar socioeconomically disadvantaged backgrounds, versus the more heterogenous backgrounds of the general population. More research is needed to explore this further.
5. Potential racial differences in the manifestation of psychological distress needs further research, as this could have implications for both the under-detection and under-treatment of mental health concerns for black FCIPs.

The meta-regression indicated fewer mental health concerns among Black FCIPs than non-Black FCIPs, and this was especially true in more recent studies. This is generally consistent with what has been deemed a race paradox in mental health (Erving et al., 2019), in which Black children and adults exhibit better mental health than would be predicted given heightened exposure to poverty, discrimination, and other stressors (Kysar-Moon, 2020; Williams, 2018) and heightened rate of physical health problems. Although differences in familial or social relationships have often been cited as a possible explanation, research evidence does not find support (Mouzon, 2013, 2014). Further, given that FCIPs tend to have weakened family relationships overall, reflecting both periods of separation and the effects of child maltreatment on relationship quality and attachment, social ties do not appear to be a viable explanation. Of note, a recent systematic review found consistently higher overall psychological distress among Black individuals than Whites despite lower rates of related conditions such as depression (Barnes & Bates, 2017). This discrepancy may point to racial differences in the manifestation of psychological distress, resulting in possible under-detection––and thus under-treatment––of mental health concerns for Black individuals.

## Supplemental Material

Supplemental Material - A Meta-Regression of Racial Disparities in Wellbeing Outcomes During and After Foster Care Click here for additional data file. Supplemental Material for A Meta-Regression of Racial Disparities in Wellbeing Outcomes During and After Foster Care by Reeve S. Kennedy, Marina H. Potter, and Sarah A. Font in Trauma, Violence, & Abuse

## Notes

1 We did not include the education/employment domain in the intercept only analysis, as it did not include enough effect sizes per racial comparison. However, it is included in the pooled positive outcomes domain that is included in the bivariate analysis.

2 Repeated datasets were the Casey National Foster Care Alumni study [10 studies] (Garcia et al., 2012, 2015; Harris et al., 2009, 2010; Harrison-Jackson, 2009; Ober, 2008; O’Brien et al., 2010; Villegas et al., 2014; Villegas & Pecora, 2012); the Midwest Evaluation of the Adult Functioning of Former Foster Youth [8 studies] (Ahrens et al., 2013; Bost, 2008; Courtney & Hook, 2017; Hook & Courtney, 2011; Lee et al., 2015; Okpych, 2017; Okpych & Courtney, 2014; Salazar et al., 2011); National Survey of Child and Adolescent Wellbeing (NSCAW) I [5 studies] (Bellamy, 2008; James et al., 2012; Leonard & Gudiño, 2016; Lloyd & Barth, 2011; Rubin et al., 2007); NSCAW II [3 studies] (Conn et al., 2014; McClellan, 2020; Xu et al., 2020); National Youth in Transition Database alone or in combination with the Adoption and Foster Care Analysis and Reporting System [9 studies] (Cheatham et al., 2020; Huang et al., 2021; Kim et al., 2019; Liu, 2020; Prince et al., 2019; Rosenberg & Kim, 2018; Shpiegel & Cascardi, 2018; Sneddon, 2019; Watt & Kim, 2019) and the Screening Impact on Services and Costs for Foster Children [SISC; 2 studies] (Taussig, 2002; Taussig & Clyman, 2011). The remaining 33 datasets were used by only one study each (Barnow et al., 2015; Calix, 2009; Chapman et al., 2014; dosReis et al., 2001; Garza-Higgens, 2011; Greeson, 2009; Harpin et al., 2013; Hill, 2010; Hindt et al., 2020; Huang et al., 2016; Huffhines et al., 2020; Jewell et al., 2010; Jonson-Reid et al., 2007; Kothari et al., 2021; Leon & Dickson, 2019; Lesperance, 2018; Milum, 2011; Munson & McMillen, 2009; Narendorf et al., 2016; Orgel, 2007; Pelnick, 2000; Reynolds et al., 2018; Ryan et al., 2010; Schmidt, 2015; Shin, 2003; Shook et al., 2013; Shovali et al., 2020; Shpiegel & Simmel, 2016; Somers et al., 2020; Theiss, 2010; White et al., 2018; Yampolskaya et al., 2011; Zima et al., 2000).
