# Supplementary material for: A Meta-Regression of Racial Disparities in Wellbeing Outcomes During and After Foster Care
Source: Trauma Violence Abuse. 2022 Jun 30;24(4):2711–25. doi: 10.1177/15248380221111481 (PMC10486179; doi:10.1177/15248380221111481)
Supplement: Supplemental Material - A Meta-Regression of Racial Disparities in Wellbeing Outcomes During and After Foster Care [file sj-pdf-1-tva-10.1177_15248380221111481.pdf]

Appendix A. Methods

Table 1. Summary of Studies

Authors (Year)	Dataset	Sample Size	% White	% Black	% Hisp.	Racial comparisons	Base-line year	Out-come year	Domains	Outcomes
Ahrens et al. (2013)	Midwest	732	31%	57%	0%	Black vs Non-Black<br>Black vs White	2002-2003		Mental Health<br>High Risk Beh.<br>Sexual Behavior	Depression symptoms<br>PTSD symptoms<br>Substance related symptoms<br>Oppositional or delinquent<br>Inconsistent condom use<br>Five or more sex partners<br>Sex for money<br>STI diagnosis-partner or self
Barnow et al., (2015)	Transition Program	1058	10%	71%	14%	Black vs White<br>Hispanic vs. White<br>Other vs. White			Employ/Earnings<br>Education	Employment<br>GED/HS<br>Post secondary education
Bellamy (2008)	NSCAW 1	604	40%	47%	17%	Black vs White	1999-2001	2002-2004	Mental Health	Internalizing
Bost (2008)	Midwest	647	35%	65%	0%	Black vs White	2002-2003	2002-2003	High Risk Beh.<br>Mental Health	Serious physical fight<br>Hurt someone badly<br>Group fight<br>Major depression<br>Alcohol dependency<br>PTSD

Calix (2009)	NC Data	1633	44%	49%	0%	Black vs White<br>Black vs Other	1999, 2000, 2001, 2002	1999, 2000, 2001, 2002	Education	Math test scores
Chapman (2016)	MHSUYLFC	406	44%	50%	1%	Black vs White		2001-2003	Mental Health	Depressive symptoms<br>Perceived stress
Cheatham et al. (2020)	NYTD, AFCARS	7117	42%	28%	21%	Black vs White<br>Hispanic vs. White<br>Other vs. White		2014-2018	Education	High school completion<br>Employment or post-secondary engagement*
Conn et al., (2014)	NSCAW II	134	55%	20%	20%	Black vs White<br>Hispanic vs. White<br>Other vs. White		2009-2011	High Risk Beh.<br>Mental Health	Depression<br>Drug Abuse<br>Loneliness
Courtney et al., (2017)	Midwest	650	29%	55%	0%	Black vs WhiteOther vs White	2002-2003	2010-2011	Education	Educational Attainment
dosReis et al. (2001)	Medicaid Insurance Claims	310			0%	Black vs White<br>Other vs White	1996	1996	Mental Health	Mental Health Services<br>Mental Disorders<br>Psychotherapeutic Medication Use
Garcia et al. (2015	Casey	805	67%	17%	15%	Hispanic vs White<br>Black vs White	2000-2002	2000-2002	Mental Health	Mental health diagnosis
Garcia et al. (2012)	Casey	805	67%	17%	15%	Hispanic vs White<br>Black vs White	2000-2002	2000-2002	Education<br>Employ/Earnings<br>Mental Health	Received diploma or GED<br>Labor Force Participation<br>One mental health disorder
Garza-Higgens (2011)	Texas educ	211	46%	38%	15%	Hispanic vs White<br>Black vs White	2008-2009	2008-2009	Education	High school completion
Greeson (2009)	Add Health	339	74%	0%	3%	POC vs White	1994-1995	2001-2002	Education<br>Employ/Earnings<br>Mental Health	Depression<br>Delinquency & violence<br>Low material hardship (higher is better)
Harpin et al. (2013)	Minnesota Student Survey	5516	54%	8%	6%	POC vs White	2007	2007	Mental Health	Emotional distress

Harris et al. (2010)	Casey	708	81%	19%	0%	Black vs White	2000-2002	2000-2002	Mental Health<br>High Risk Beh.	Major depression diagnosis in last 12 months<br>Oanic syndrome diagnosis in last 12 months<br>Modified social phobia diagnosis in last 12 months<br>Generalized anxiety diagnosis in last 12 months<br>PTSD diagnosis in last 12 months<br>Drug dependence in last 12 months
Harris et al. (200()	Casey	708	81%	19%	0%	Black vs White	2000-2002	2000-2002	Education<br>Employ/Earnings<br>Homelessness	Completed high school with diploma or GED<br>Completed high school with GED<br>Any post-secondary education<br>Any completed post-secondary education<br>Bachelor's degree<br>Never received public assistance since turning 18<br>Not receiving public assistance at the time of the interview<br>No one in household has received assistance in the past 6 months<br>Income at or above poverty<br>Income greater than three time poverty<br>Does not own house or apartment<br>Homeless within a year of leaving care<br>Currently employed
Harrison-Jackson et al. (2009)	Casey	1582	36%	8%	7%	Black vs White	2000-2002	2000-2002	Education	Years of education
Hill (2011)	Minn_Link<br>Minnesota Admin	2187	59%	22%	6%	Hispanic vs White<br>Black vs White<br>AI/AN vs White<br>Asian vs White	2002-2004	2006-2008	Criminal<br>Education	Adult corrections (probation or parole)<br>Did not drop out of high school

Hindt et al. (2020)	RKCP	274	7%	66%	13%	Black vs Non-Black			High Risk Beh.<br>Mental Health	Externalizing<br>internalizing
Hook et al. (2011)	Midwest	732	27%	58%	9%	Black vs White	2002-2003	2008-2009	Employ/Earnings	Employed<br>Employed 20+ hours
Huang et al. (2021)	NYTD AFCARS	4853	39%	28%	24%	Black vs White<br>Hispanic vs. White<br>Other vs. White	2014	2018	Homelessness<br>Criminal	Homelessness<br>Incarceration
Huang et al. (2016)	Illinois Admin	145	2%	92%	6%	Black vs Hispanic	1994		Criminal	Time to juvenile arrest
Huffhines et al. (2020)	SPARK	283	31%	40%	9%	POC vs White			Mental Health	Self-reported internalizing<br>Caregiver-reported internalizing
Jackson Foster et al. (2015)	Casey	1038	72%	8%	11%	Black vs White<br>AI/AN vs White<br>Hispanic vs White	2000-2002	2000-2002	Mental Health	Psychiatric Comorbidity
James et al. (2012)	NSCAW 1	1191	41%	39%	15%	Black vs White<br>Hispanic vs. White<br>Other vs. White	1999-2001	2002-2004	High Risk Beh.<br>Mental Health	Total behavior problems score<br>Externalizing behavior score<br>Internalizing behavior score
Jewell et al. (2010)	Family home program	427	76%	24%	0%	Black vs White	2000-2003	2000-2003	High Risk Beh.	Aggression Problem behaviorsSchool problems
Jonson-Reid et al. (2007)	MCD	339	44%	0%	0%	POC vs White	2001-2003	2001-2003	High Risk Beh.	Dating violence Perpetration
Kim et al. (2019)	NYTD AFCARS	4206	45%	30%	17%	Black vs White<br>Hispanic vs. White<br>Other vs. White	2011	2013, 2015	Education<br>Employ/Earnings	High school completion<br>Post secondary education<br>Employment
Kothari et al. (2020)	SIBS-FC	315	51%	0%	0%	POC vs White	2009-2013	2009-2013	Education	Math educational resilience<br>Reading educational resilience<br>School Attendence
Lee et al. (2015)	Midwest	732	29%	55%	0%	Black vs White<br>Other vs White	2002-2003	2006-2009	Criminal	Criminal activities at 21<br>Criminal activites at 23 or 24

Leon & Dickson (2019)	Finding Family	221	0%	58%	14%	Black vs Non-Black	2011-2014	2011-2014	High Risk Beh.<br>Mental Health	Internalizing<br>Externalizing
Leonard & Gudino (2016)	NSCAW 1	420	44%	36%	11%	Black vs White<br>Hispanic vs. White<br>Other vs. White	1999-2001	2002-2004	Education<br>High Risk Beh.<br>Mental Health	Reading Achievement<br>Math Achievement<br>Internalizing<br>Externalizing
Lesperance (2018)	Detroit age out	57	25%	0%	0%	POC vs White	2005-2006	2009-2011	Criminal	Spent time in jail lifetime<br>Charged or convicted of offense
Lloyd & Barth (2011)	NSCAW 1	353	40%	31%	21%	POC vs Non-POC	1999-2001	2005-2007	Education	WJ: letters<br>WJ; passages<br>WJ: applied math
Lui (2020)	NYTD	5633	39%	28%	24%	Black vs White<br>Hispanic vs White<br>Asian vs White<br>AI/AN vs White<br>Native Hawaiian/Alaska Native vs White<br>Multi-Racial vs White	2014	2018	Education<br>Employment<br>Homelessness	Educational Attainment<br>Employment<br>Homelessness
McClellan (2020)	NSCAW II	296	40%	39%	0%	Black vs White	2008-2009	2011-2012	Mental Health	Internalizing
Milum (2011)	ACAILP	66	73%	0%	9%	POC vs White	2005-2009	2005-2009	EducationEmployment	High School Degree/GEDEmployed
Munson (2009)	Missouri youth transitioning	339	45%	52%	0%	POC vs White	2001-2003	2003-2005	Mental Health<br>Employment<br>High Risk Beh.<br>Criminal	Depression symptoms at19<br>Perceived stress at 19<br>Currently employed<br>Past year alcohol use<br>Past year marijuana use<br>Arrest

Narendorf et al. (2016)	Missouri children division	325	41%	0%	0%	POC vs White	2001-2003	2003-2005	Education<br>Mental Health<br>Homelessness<br>Sexual Behavior<br>Employ/Earnings<br>Criminal	Skills deficits (higher is worse)<br>Affect instability (higher is worse)<br>In outpatient therapy<br>Taking psychotropic medications<br>Psychiatric hospitalizations<br>Residential treatment<br>Homelessness<br>High risk sex<br>Employed<br>Graduated high school<br>Arrest
O'Brien et al. (2010)	Casey	817	70%	0%	0%	AI/AN vs White	2000-2002	2000-2002	Education<br>Employ/Earnings<br>Homelessness	Diploma or GED<br>Diploma<br>GED<br>Any education past high school<br>Completed any degree/certificate past high school<br>Completed college or more<br>Employment in workforce<br>Has health insurance of any kind<br>Homeless for 1 or more nights since leaving care<br>Does not own house or apartment<br>household income above poverty line<br>Household income greater than 3x the poverty line<br>never received any public assistance since turning 18<br>Not receiving public assistance at the time of the interview<br>No one in household has received assistance in the past 6 months

Ober (2008)	Casey	1068	51%	13%	12%	Black vs WhiteHispanic vs. WhiteOther vs. White	2000-2002	2000-2002	Mental Health	Alcohol Dependence in last 12 months
Okpych (2017)	Midwest	732	29%	55%	9%	Black vs White<br>Hispanic vs. White<br>Other vs. White	2000-2003	2010-2011	Education	College entry<br>College persistence<br>Creditional completion<br>Degree completion
Okpych & Courtnery (2014)	Midwest	564	36%	60%	0%	Black vs White<br>Other vs. White	2000-2003	2010-2011	Employ/Earnings	Likelihood of employment
Orgel (2007)	Children Assessment Service (CAS)- Portland Archival	127	59%	12%	4%	Black vs Non-Black<br>Hispanic vs Non-Hispanic<br>POC vs White<br>Multi-Racial vs Non-Multi-Racial	1996-2003	1996-2003	High Risk Beh.<br>Mental Health	Total behavior problems score<br>Internalizing<br>Externalizing
Pelnick (2000)	Syracuse Admin	69	19%	65%	4%	Black vs Non-Black	1990	2000	Education	School disciplinary actionGPA
Prince (2019)	NYTD<br>AFCARS<br>ACS	7449	30%	19%	12%	Black vs White<br>Hispanic vs. White<br>Other vs. White	2011	2013	Homelessness<br>Criminal<br>Sexual Behavior	Homelessness<br>Incarceration<br>Substance abuse<br>Child birth
Reynolds et al. (2018)	Voluntary trans living program	2913	49%	40%	6%	Black vs White<br>Hispanic vs. White<br>Other vs. White	2010	2014	Homelessness	Housing Insecurity
Rosenberg & Kim (2018)	NYTD<br>AFCARS	4235	45%	30%	17%		2011	2015	Education	Post secondary education<br>Post secondary education and/or fulltime employment*

Rubin et al. (2007)	NSCAW 1	729	44%	38%	13%	Black vs White<br>Hispanic vs. White<br>Other vs. White<br>Multi-Racial vs White	1999-2000	2001-2002	High Risk Beh.	Abnormal behavioral wellbeing
Ryan et al. (2010)	LA DCFS	13396	17%	29%	51%	Black vs White<br>Hispanic vs. White<br>Asian vs White	2002-2008	2002-2008	Criminal	Arrest
Salazar et al. (2011)	Midwest	513	33%	55%	0%	Black vs White<br>POC vs White	2002-2003	2006-2007	Mental Health	Depressive symptom count
Schmidt (2015)	My Life	294	42%	0%	0%	POC vs White			Employ/Earnings	Employment
Shin (2003)	Illinois DCFS	152	28%	64%	4%	POC vs White	1998	1998	Education	Reading achievement
Shook et al. (2013)	PA Admin	4275	34%	59%	0%	Black vs Non-Black	2002-2008	2002-2008	Criminal<br>Mental Health<br>High Risk Beh.	Juvenile Justice involvement<br>Jail<br>Mental health System Involvement<br>Drugs and Alcohol
Shovali et al. (2020)	NSCNC	809	53%	22%	12%	Hispanic vs Non-Hispanic	2013		Education	Math achievement<br>Reading/writing achievement
Shpiegal et al. (2018)	NYTD	3173	43%	30%	18%	Black vs White<br>Hispanic vs. White<br>Other vs. White	2011	2015	EducationEmploy/EarningsHomelessnessMental HealthCriminal	Diploma/GED or aboveCurrently employedNot receiving public assistanceHomelessnessSubstance abuse referralIncarceration
Shpiegal et al. (2016)	MSEYP	405	33%	44%	43%	POC vs White<br>Hispanic vs Non-Hispanic	2003-2007	2005-2009	Education<br>Employ/Earnings<br>Homelessness	Diploma/GED<br>Employment<br>Homelessness<br>Financial Stability

Sneddon (2019)	NYTD AFCARS	3968	57%	33%	12%	Black vs White<br>Hispanic vs. Non-Hispanic<br>Other vs. White	2011	2015	Homelessness	Risk of homelessness
Somers et al. (2020)	Fostering Healthy Futures	363	48%	24%	46%	Hispanic vs Non-Hispanic<br>AI/AN vs White<br>Black vs Non-Black<br>POC vs White<br>Other vs White, Black, Hispanic, American Indian	2007-2011	2007-2011	Education<br>High Risk Beh.	Academic achievement<br>Detention/suspension
Taussig (2002)	SISC	110	44%	36%	20%	Black vs Hispanic & White<br>Hispanic vs White	1990-1991	1995-1996	High Risk Beh.<br>Sexual Behavior	Delinquency<br>Self-destructive behavior<br>Substance abuse<br>Sexual risk behavior<br>Total risk behavior
Taussig & Clyman (2011)	SISC	149	42%	32%	20%	Black vs Non-Black	1990-1991	1995-1996	High Risk Beh.<br>Sexual Behavior<br>Criminal	Delinquency<br>Sexual risk behavior<br>Substance Use<br>Tickets/Arrests<br>Suspensions<br>behavior problems
Theiss (2010)	Ohio admin	243	46%	54%	0%	Black vs White	2008-2009	2008-2009	Mental Health	Mental health diagnosis
Villegas & Pecora (2012)	Casey	810	71%	15%	14%	Black vs WhiteHispanic vs White	2000-2002	2000-2002	Mental Health	Mental health diagnosis

Villegas et al. (2014)	Casey	810	71%	15%	14%	Black vs White<br>Hispanic vs White	2000-2002	2000-2002	Education	High school diploma or GED<br>GED<br>High school diploma<br>Post-high school certificate<br>Bachelor's degree or higher
Watt & Kim (2019)	NYTD	9342	45%	31%	16%	Black vs White<br>AI/AN vs White<br>Hispanic vs White<br>Other vs White	2011	2015	Education<br>Employ/Earnings<br>Homelessness<br>Criminal	High school diploma or GED<br>Enrolled in higher education<br>Employed ft/pt<br>Homeless<br>Incarceration
White et al. (2018)	VOYAGES	312	43%	57%	0%	Black vs White	2001-2003	2003-2005	Education	Graduated high school<br>Received GED<br>Started college
Xu et al. (2020)	NSCAW II	263	33%	40%	28%	Black vs White<br>Hispanic vs White	2011-2012		Mental Health<br>High Risk Beh.	Internalizing<br>Externalizing
Yampolskaya et al. (2011)	Florida admin	13212	56%	36%	8%	Black vs White<br>Hispanic vs White	2003-2004	2005-2006	Criminal	Juvenile detention facility placement
Zima et al. (2000)	Los Angeles DCFS	302	19%	33%	37%	Hispanic vs White	1996-1998		High Risk Beh	At least one behavior problem

Appendix B. Study Selection and Coding

Table 1. Coding Scheme – All Variables except domain

**Study Level**	**Response Options**	**Description**
id	#	Each data entry receives a number, 1, 2, 3, …
studyid	#	each study receives a number, 2001, 2003, 2003, …
author	*Name, et al.*	Study authors names – follow APA for

		citations
year	*Year*	Publication year
pubtype	*Peer, diss, report*	Type of publication: peer-reviewed journal article, dissertation, report or policy brief
design	*Long, cross, rct, quasi, admin, evaluation*	Type of research design: longitudinal, cross-sectional, randomized controlled trial, quasi-experimental, evaluation
sampling	*Random, nonrandom*	Type of sampling – random sample or non-random sample
dataset	*name*	Name of dataset – if no name is provided – assign a brief descriptive name
baseline_datayear	*Baseline year(s)*	When was baseline data collected
baseline_recode	*Baseline starting year*	What year did baseline data collection start
baseline_length	*# (years)*	How long (years) was baseline data collection
outcome_datayear	*year*	When were the outcomes collected
outcome_recode	*year*	What was the last year outcome data was collected
outcomeyear_length	*# (years*	How long (years) was the outcome data collected
state	*State name, multi-state*	Which state was the sample collected in?
region	*West, Midwest, north east, south, national*	Using census groupings, in which region of the US was the data collected
location	*location*	Location of the data collection
datatype	*Interview, admin, survey, etc*	Type of data - interviews, administrative, survey data
datasource	*Self, caregiver, teacher, admin*	Who is providing the data? Self-report, caregivers, teachers, official administrative data
**Effect Size Details**		

outcome	*Externalizing, internalizing, math scores, depression symptoms, etc*	The specific indicator provided in the study. Many studies will have multiple indicators, each one is its own line of data (with a new ID, but the same study ID)
domain	*Behavior, housing, employment/earnings, criminal behavior, etc*	The broader domain the indicator falls into. See table below for more detail about indicators and domains
es_type	*OR, regression, mean, chi square p, etc*	What type of data is being extracted from the study to calculate the effect size
covariance	*Yes, no*	Is the effect size part of a model that controls for other variables
outcome_race	*Black, White, Hispanic, etc*	The primary racial group being examined for the outcome
outcome_ref	*White, non-white, Black, not-Black, etc*	The reference or comparison racial group for the outcome
OR	# (2 decimal)	Odds ratio - either extracted directly from paper or calculated
orci_lower	# (2 decimal)	95% confidence interval lower limit of OR, either from study or calculated
orci_upper	# (2 decimal)	95% confidence interval upper limit of OR, either from study or calculated
stdmdiff	# (2 decimal)	Standardized mean difference/Cohen's d – either directly from study or calculated
mci_lower	# (2 decimal)	Standardized mean difference 95% confidence interval lower limit
mci_upper	# (2 decimal)	Standardized mean difference 95% confidence interval lower limit
se_	# (2 decimal)	Standard error – typically extracted directly from study
n_outcome	#	N value for the outcome measure (often not the same as the sample N)
**Data Details**		

status_datacollect	*In care, out of care, mixed*	Average placement status at time of data collection – in care, out of care, mixed
majority_pt	*Foster, kinship, etc*	What type of placement are/were the majority of the participants in? Ex – foster care, kinship care, group care, residential care
age_entry	#	Average age at entry to foster care
type_exit	*Age out, adoption, reunification*	What is the average type of exit for the sample? Ex – age out, reunification, adoption
avg_lengthstay	<1 year, 1-2 years, etc	length of stay (mode – most common in sample)
avg_placements	#	Average number of placements
illtrans_services	*Yes, no, (or missing)*	Did the participants receive independent living or transitional living serves?
**Participant Level**		
mean_age	#	Mean age of sample
mean_age_recode	#	Mean age of sample or median if age was a range
sample_n	#	Whole sample N
white_n	#	Number of white participants
black_n	#	Number of black or African American participants
hispanic_n	#	Number of Hispanic participants
asian_n	#	Number of Asian participants
multiracial_	#	Number of biracial or multi-racial (self identified) participants
nativehawaiipacific_n	#	Number of native Hawaiian or pacific islander participants
nh_pi_asian_n	#	Number of Native Hawaiian, pacific islander, asian participants
ai_na_n	#	Number American Indian or Native

		Alaskan participants
unknown_n	#	Number of participants of unknown race
other_notwb_n	#	Number of other race, excluding White and Black (includes Hispanic) participants
other_notwbh_n	#	Number of other race, excluding White, Black, and Hispanic participants
male_n	#	Number of male participants
female_n	#	Number of female participants
Updated Variables		
outcome_race_final	*Black, POC, etc*	Inverted (if necessary) outcome race
outcome_ref_final	*White, non-Back, etc*	Inverted (if necessary) outcome reference group
or_final	#	Updated OR (inverted if necessary)
or_ll_final	#	Updated 95% CI lower limit (inverted if necessary)
or_ul_final	#	Updated 95% CI upper limit (inverted if necessary)
comparison	*Black vs White, etc*	OR comparisons (second group is reference group)

Search Terms

To locate all studies that addressed outcomes of foster care by race/ethnicity, the lead author conducted a systematic search of PsycINFO, ERIC, and Academic Search Complete using a series of search term combinations. These search terms addressed the type of placement, including "foster care," "kinship care," "out of home care," "congregate care," "foster youth," "foster child*, and "ag* out." These terms were separated by "OR" and were combined with terms related to specific types of outcomes and were connected with an "AND". Outcome terms included, "behavior* problem" or "externalizing" or "internalizing," "mental health" or "depress*," "crim*" or "delinq*," educat*" or "academic achievement," "housing" or "homeless" or "housing stability," "earning*,""employ*," "pregnan*" or "teen parent" or teen moth*," "drug use" or "substance use" or "substance abuse" or "drug abuse". Finally, the term "outcome" was included in all search phrases with "AND".

Exclusions during study selection

Two hundred and thirty-five studies were excluded during full-text review. The most common reasons (only the primary reason for exclusion was recorded) for exclusion during the full-text review were: not including outcomes by race (n = 124), not focusing on the general population of care-as-usual (n = 55), having a study design that did not focus on the analysis of outcomes (n = 13), and focusing on outcomes that were not the focus of this review (n = 12). Other reasons for full-text exclusion that included fewer than 10 studies each were: not enough information to calculate effect size (n = 6), dissertation duplicate (n = 7), non-US (n = 6), data duplicate with missing values or poor fit (n = 4), only one racial/ethnic group (n = 3), missed duplicate (n = 1), wrong publication type (n = 1), and policy brief duplicate (n = 1). There were 87 studies after full-text review that met the inclusion criteria. During data extraction, 16 additional studies were excluded. The most common reason for exclusion during data extraction was for not providing enough information to calculate an effect size (n = 12), followed by wrong population (n = 2) and not enough info about the racial groups (n=2).

Coding

Inter-rater Agreement. To develop inter-rater agreement, coders completed ten studies, then compared the extracted data. This was repeated three times before proceeding with the full sample. The overall inter-rater agreement for the whole sample was 79% (including early rounds).

Adjustments to data. If a study reported effect sizes separated by relevant populations (e.g. heterosexual and homosexual), the two effect sizes were combined and averaged (Rosenthal, 1991; Tsaousis, 2016). This was done for three studies (Chapman et al., 2014; Hook & Courtney, 2011; Jewell et al., 2010). For missing data, we followed the steps outlined by Pigott and Polanin (Pigott & Polanin, 2020) and we inferred from the study where possible and contacted authors if necessary. We contacted two authors who included all of the necessary information for the effect size, but failed to define what race meant in their models (Huffhines et al., 2020; Strong-Blakeney, 2013)– we heard back from one of the authors and the study was included in the analysis. For moderators, we chose to leave values as missing if it the information was not available. Finally, we focused on outcomes that fell within seven different domains (sexual health, mental health, homelessness, high risk behavior, employment, education/earnings, criminal behavior). To do this, we combined multiple indicators/variables that assessed similar concepts that fall within each broader domain, which is similar to the approach taken by other published meta-analyses (Dam et al., 2018; DuBois et al., 2011; Eby et al., 2013).

We originally had an additional domain: employment/education, that included effect sizes that were assessing educational achievement or employment status. This domain only had seven effect sizes that were from two studies. These two studies (Cheatham et al., 2020; Rosenberg & Kim, 2018) had other effect sizes in the Education domain. We decided to drop the employment/education domain because it was too small for analysis by racial comparison group and the studies were already being represented in the education domain. These effect sizes, however, are included in the overall positive outcome analysis.

When a study had an outcome that was in the opposite direction of the others within its domain (e.g. Greeson (2009) looked at material hardship with a higher value representing more material hardship, while the other measures in the earnings/employment domain had higher values representing more positive outcomes, such as being employed and amount of earnings), if available, the standard mean difference was multiplied by -1, then the odds ratio was calculated from the new SMD. This was also done with odds

ratios. For two studies, we inverted the odds ratios so the scale was consistent with the others in the domain (Hill, 2010; Shpiegel & Cascardi, 2018).The domains were then grouped into positive outcomes (Education, employment/ earnings) and negative outcomes (criminal behavior, high risk behavior, homelessness, mental health difficulties, and high-risk sexual behavior).

We aimed to make White the reference group for all of our race/ethnicity comparisons. For studies that did not include White or similar as the reference group, we inverted the odds ratios to move White or similar (e.g. non-Black or non-Hispanic) to the reference group. This was done for eight difference studies (Calix, 2009; Greeson, 2009; Milum, 2011; Orgel, 2007; Shin, 2003; Shpiegel & Simmel, 2016; Somers et al., 2020; Zima et al., 2000). For studies that were extracted as frequencies or binary proportions, we reversed the direction of the scale to put White or similar as the reference group. This was done for five studies (Garcia et al., 2012; Harris et al., 2009, 2010; O'Brien et al., 2010; Villegas et al., 2014).

Appendix C. Additional Results

Sensitivity Tests

For the first sensitivity test we dropped 20 effect sizes that were extremely highly correlated. For example, in Harris (2009), there is an effect size that assesses completing high school with a diploma or GED, while there are two additional effect sizes (ES) that assess completing high school with a diploma and another that assesses completing high school with a GED. For the sensitivity analyses we dropped the variable that included *both* GED and diploma. Others that were dropped looked at Total Behavior Problems (while the individual problem behaviors were assessed in other ES), public assistance receipt (we dropped all but current public assistance), and amount above poverty line (we dropped 3x over poverty line). The other sensitivity test we ran involved testing out different correlation values used for estimating the within study effect size correlation. Our primary analyses used RHO (.08): for the sensitivity tests we ran all analyses with the RHO (.07) and RHO (.09). These alternative values had little to no difference on the estimates.

Publication Bias

In addition to the full sample, we conducted funnel plots and egger regression tests for each racial comparison/domain combination due to anticipated high levels of heterogeneity within the whole sample. Egger tests were conducted when the models had ten or more effect sizes (Borenstein et al., 2009). For the whole sample, visual inspection of the funnel plot revealed a small amount of asymmetry, and the egger regression indicated the presence of small sample effects (beta1: -.52, SE: .175, p<.01). We suspect that much of this is due to heterogeneity within the dataset (I^2=84.62), especially given our focus on different racial comparisons within different domains. Thus, we conducted funnel plots and egger regression tests for small study effects for each racial comparison within each domain to see if we could parse out the source of the asymmetry. We found no evidence of small study effects in the majority of the racial group comparisons within each domain. However, we found evidence of small study effects for the Black vs Non-Black comparison in the education and earnings/employment domains, and thus the positive outcomes as well. Relatedly, Black

vs White had a $p < .05$ value in the employment/earnings domain (the Black vs White comparison makes up most of the Black vs Non-Black comparison, so this was expected). Hispanic vs White was significant at the $p<.05$ level for the positive outcomes and the high-risk behavior domain. Due to issues related to the file drawer effect (Rosenthal, 1979), we chose to include publication type (peer review vs dissertation) as a moderator in a second round of egger regression tests. We found that when publication type was included in the model, the evidence of small study effects disappeared. This suggests that while there was some evidence of small study effects in our sample, it was largely tied to the publication type of the study. Finally, we conducted the trim and fill method, which resulted in no imputations, suggesting low asymmetry. However, due to high degrees of heterogeneity in the sample, the trim and fill findings need to be interpreted with caution.

	Educational Achievement	High Risk & Externalizing	Mental Health Concerns	Negative Outcomes[b]	Positive Outcomes[c]

Appendix D

Full Results for Bivariate Meta-Regressions

Table 1. Bivariate Meta-Regressions with Covariates: Black vs Non-Black Comparisons[d]

	Educational Achievement	High Risk & Externalizing	Mental Health Concerns	Negative Outcomes[b]	Positive Outcomes[c]
	OR (95% CI)	OR (95% CI)	OR (95% CI)	OR (95% CI)	OR (95% CI)
Study Features					
Baseline year (base: pre-2002)					
2002+	1.16 (0.90, 1.49)	0.79 (0.52, 1.19)	**.79 (0.64, 0.97)**	0.94 (0.74, 1.19)	1.08 (0.85, 1.39)
	OR (95% CI)	OR (95% CI)	OR (95% CI)	OR (95% CI)	OR (95% CI)
Outcome year (base: pre-2008)					
Study Features	1.17 (0.91, 1.51)	0.87 (0.54, 1.42)	1.17 (0.58, 1.25)	0.95 (0.74, 1.21)	1.12 (0.88, 1.42)
2008+					
Baseline year (base: pre-2002)	1.28 (0.95, 1.73)			0.96 (0.69, 1.35)	1.38[a] (1.02, 1.86)
Data source (base: administrative)					
Survey – Random sample		0.84[a] (0.50, 1.40)	1.04[a] (0.67, 1.60)	0.97 (0.63, 1.49)	1.34[a] (0.77, 2.29)
Survey – Non-random sample		0.79[a] (0.39, 1.60)	0.76[a] (0.42, 1.36)	1.17 (0.84, 1.63)	1.20[a] (0.64, 2.23)
Effect Size Type (base: Other) Regression coefficient	1.13 (0.86, 1.48)	0.75 (0.51, 1.09)	1.03 (0.73, 1.48)	0.92 (0.73, 1.17)	1.16 (0.92, 1.46)
Self-reported outcome (base: No) Yes	1.35 (0.98, 1.88)	1.15 (0.79, 1.68)	1.02 (0.74, 1.42)	1.06 (0.83, 1.38)	1.16 (0.83, 1.63)
Publication type (base: dissertation) Peer-reviewed	**1.60 (1.15, 2.23**)	0.65[a] (0.31, 1.38)		1.11 (.69, 1.79)	**1.42 (1.02, 1.97)**
Sample type (base: regional) Nationally representative	1.22 (0.93, 1.62)	0.68 (0.45, 1.04)	0.76 (0.47, 1.23)	0.85 (0.66, 1.11)	**1.30 (1.02, 1.63)**
Sample Features					
Foster care status at outcome (base: in care)					
Out of care minor (under age 18)		0.88[a] (0.51, 1.54)	0.70[a] (0.33, 1.46)	0.73 (0.44, 1.21)	0.46[a] (0.29, 0.73)
Out of care adult	0.95[a] (0.51, 1.77)	0.85 (0.46, 1.58)	0.93 (0.66, 1.32)	0.87 (0.66, 1.15)	0.90[a] (0.48, 1.67)

[a] Degrees of Freedom < 4, significance level for df<4 is p<.001 [b] Negative domains include: high risk behavior, mental health concerns, justice system involvement, homelessness, sexual behavior [c] Positive domains include: education, education/employment, employment/earnings [d] Black vs Non-Black combines all racial comparisons that looked at Black vs another racial group, this includes Black vs White.

Bolded: statistically significant

Table 2. Bivariate Meta-Regressions with Covariates: Hispanic vs Non-Hispanic Comparisons[d]

2002+					
Outcome year (base: pre-2008)					
2008+	1.26[a] (0.92, 1.72)			0.93 (0.70, 1.22)	1.39[a] (1.03, 1.86)
Data source (base: administrative)					
Survey – Random sample				1.00[a] (0.71, 1.42)	
Survey – Non-random sample				0.76 (0.49, 1.16)	
Effect Size Type (base: Other)					
Regression coefficient	1.14[a] (0.70, 1.86)	1.00[a] (0.49, 2.03)	0.54[a] (0.31, 1.06)	**0.78** (0.61, 0.99)	1.23[a] (0.74, 2.05)
Self-reported outcome (base: No)					
Yes	1.07[a] (0.78, 1.46)	1.31 (0.73, 2.39)		1.26 (0.91, 1.75)	1.05[a] (0.79, 1.40)
Publication type (base: dissertation)					
Peer-reviewed	1.13[a] (0.14, 9.03)			1.11[a] (0.48, 2.56)	1.16[a] (0.14, 9.49)
Sample type (base: regional)					
Nationally representative	1.07 (0.76, 1.51)	1.19[a] (0.51, 2.75)	0.55[a] (0.28, 1.12)	1.19[a] (0.51, 2.75)	1.12 (0.80, 1.55)
Sample Features					
Foster care status at outcome (base: in care)					
Out of care minor (under age 18)				**1.97** (1.43, 2.75)	
Out of care adult	1.01[a] (0.66, 1.55)			1.27 (0.87, 1.86)	1.05[a] (0.70, 1.58)

[a] Degrees of Freedom < 4, significance level for df<4 is p<.001 [b] Negative domains include: high risk behavior, mental health concerns, justice system involvement, homelessness, sexual behavior [c] Positive domains include: education, education/employment, employment/earnings [d] Black vs Non-Black combines all racial comparisons that looked at Hispanic vs another racial group, this includes Hispanic vs White.
Bolded: statistically significant
